# Exploring the antibacterial potential of arthrocolins against extensively drug-resistant *Pseudomonas aeruginosa*: mechanistic insights into amino acid metabolism disruption

**DOI:** 10.3389/fmicb.2025.1618419

**Published:** 2025-09-23

**Authors:** Bao-Rui Yang, Yun-Wen Zhang, Qun-Fu Wu, Jian-Mei Liu, Guang-Juan Wu, Wei-Juan Tian, De-Yao Deng, Xue-Mei Niu, Wen-Li Yuan

**Affiliations:** ^1^Department of Clinical Laboratory, The Affiliated Hospital of Yunnan University (The Second People's Hospital of Yunnan Province), Kunming, China; ^2^Laboratory for Conservation and Utilization of Bio-Resources, Key Laboratory for Microbial Resources of the Ministry of Education, Yunnan University, Kunming, China

**Keywords:** extensively drug-resistant *Pseudomonas aeruginosa*, arthrocolins, the antibacterial effect, transcriptomics, metabolomics

## Abstract

**Introduction:**

Extensively drug-resistant Pseudomonas aeruginosa (XDR-PA) poses a serious clinical threat due to its intrinsic resistance mechanisms and the lack of effective therapeutic agents. This study aimed to evaluate the antibacterial activity of arthrocolins (Acs), a novel group of xanthene-like compounds isolated from Escherichia coli, against XDR-PA.

**Methods:**

Clinical data analysis was conducted to identify significant risk factors for XDR-PA infection. Drug susceptibility testing was performed to assess the effectiveness of Acs. Transmission electron microscopy (TEM) was used to observe cellular changes in Acs-treated bacteria, while integrated transcriptomic and metabolomic analyses were employed to investigate the underlying mechanisms.

**Results:**

Acs demonstrated significant antibacterial activity, inhibiting XDR-PA growth at low micromolar concentrations. The IC50 value was determined to be 3.094 μM. Clinical data analysis identified prolonged antimicrobial therapy, invasive procedures, and extended hospitalization as significant risk factors for XDR-PA infection. TEM revealed cell wall disruption and cytoplasmic condensation in Acs-treated bacteria. Integrated omics analyses indicated that Acs interferes with amino acid metabolism, impairing energy production and causing abnormal lipid accumulation.

**Discussion:**

These findings suggest that Acs exerts potent antibacterial effects through disruption of metabolic homeostasis and structural integrity. The study highlights the potential of Acs as a promising candidate for the treatment of XDR-PA infections, offering a new avenue for therapeutic development. The bacterial names cannot be italicized in the annotations. The revised abstract has been uploaded separately as an attachment.

## Introduction

According to the 2024 WHO bacterial priority pathogens list, carbapenem-resistant *Pseudomonas aeruginosa* (CRPA) remains among the most critical threats due to its high prevalence of multidrug- and extensively drug-resistant (MDR/XDR) phenotypes and the scarcity of effective treatment options (World Health Organization, 2024). A recent comprehensive review further summarized the major resistance mechanisms—including β-lactamase production, efflux pump activity, porin loss, and adaptive regulatory changes—and outlined emerging therapeutic approaches, thereby underscoring the urgent need for novel interventions (Elfadadny et al., 2024).

Epidemiological investigations consistently demonstrate a substantial burden of *P. aeruginosa* infections worldwide. High colonization, infection, and recurrence rates have been documented in respiratory specimens (de Souza Nunes et al., 2023), and increasing MDR/XDR prevalence has been reported across different clinical isolates (Kothari et al., 2023; Buhl et al., 2015). Multicenter and regional surveys from both Asian and Western countries corroborate these findings and identify healthcare exposure and prior antibiotic use as key contributors to resistance development (Palavutitotai et al., 2018; Saleem and Bokhari, 2019; Hosseininassab Nodoushan et al., 2017; Pereira et al., 2014; Park et al., 2011; Willmann et al., 2014; Kang et al., 2021; Samonis et al., 2014; Karlowsky et al., 2005).

The clinical consequences are particularly severe in high-risk populations. Hospitalized patients, burn victims, children, and individuals with hematological malignancies or cancer are disproportionately affected by MDR/XDR *P. aeruginosa*, with increased morbidity and mortality (Palavutitotai et al., 2018; Saleem and Bokhari, 2019; Hosseininassab Nodoushan et al., 2017; Pereira et al., 2014; Park et al., 2011; Willmann et al., 2014; Kang et al., 2021; Samonis et al., 2014; Karlowsky et al., 2005). Such evidence highlights the urgent necessity of integrating effective infection-control measures with the development of innovative antimicrobial strategies.

Polymyxins, particularly colistin, have long been regarded as last-resort agents; however, their clinical utility is constrained by severe nephrotoxicity (Zavascki and Nation, 2017) and inconsistent treatment outcomes (Montero et al., 2009). Moreover, adaptive resistance mediated by two-component regulatory systems has been described (Fernández et al., 2010), and resistant isolates continue to emerge in diverse settings (Goli et al., 2016; Abd El-Baky et al., 2020). Although newer β-lactam/β-lactamase inhibitor combinations such as ceftolozane–tazobactam have expanded treatment options, susceptibility remains heterogeneous across resistance backgrounds (Shortridge et al., 2018). These limitations collectively highlight a persistent therapeutic gap.

Fluorescein-like arthrocolins (Acs) represent a recently identified family of natural products that can be biosynthesized in engineered *Escherichia coli* (Chen et al., 2019). Beyond antibacterial interest, Acs have shown synergistic activity with fluconazole against resistant *Candida albicans* by enhancing riboflavin metabolism and inducing mitochondrial dysfunction and autophagy (Wu et al., 2023). These findings suggest that Acs possess multifaceted bioactivity and warrant evaluation as potential antibacterial agents.

In this study, we investigated the antibacterial activity of Acs against an XDR *P. aeruginosa* clinical isolate, characterized concentration–response relationships, and explored the underlying mechanism with a particular focus on amino acid metabolism and related pathways highlighted by our omics analyses. These results provide mechanistic insights into metabolic vulnerabilities that could inform the development of novel therapeutic strategies.

## Materials and methods

### Materials

Arthrocolins (Acs) were provided by Laboratory for Conservation and Utilization of Bio-Resources, Key Laboratory for Microbial Resources of the Ministry of Education, Yunnan University with a purity greater than 95%. Dimethyl sulfoxide (DMSO) was purchased from Solarbio (China). Drug susceptibility testing was conducted using the VITEK 2 COMPACT system (bioMérieux, France) following the guidelines of the Clinical and Laboratory Standards Institute (CLSI, M100, 32nd Edition, 2022). Quality control was ensured using the reference strain *P. aeruginosa* ATCC 27853.

A total of 228 non-duplicated extensively drug-resistant *P. aeruginosa* (XDR-PA) isolates were collected from hospitalized patients at Affiliated Hospital of Yunnan University Hospital. Bacterial identification was confirmed by 16S rRNA sequencing. Patient data collection and bacterial isolation were approved by the institutional ethics committee (approval number: 2023009), and written informed consent was obtained from all participants. Among these isolates, one representative strain with the MLST subtype ST3390, isolated from the urine of a hospitalized patient, was selected for further studies, including IC_50_ determination, TEM observation, transcriptomic sequencing, and metabolomic profiling.

### Methods

Based on the materials described above, a series of experimental procedures were conducted to evaluate the antibacterial activity of arthrocolins (Acs) and to investigate their underlying mechanisms against XDR-*P. aeruginosa*. These procedures included drug susceptibility testing, IC_50_ determination, transmission electron microscopy (TEM), transcriptomic sequencing, and metabolomic profiling. All experiments were performed following standard protocols, and each test was carried out in triplicate unless otherwise specified.

#### Detection and collection of clinical XDR-PA strains

We investigated the drug resistance profile of *P. aeruginosa* (PA) isolated from clinical specimens of patients treated at the Affiliated Hospital of Yunnan University from December 2018 to June 2023 and collected information on some hospitalized patients. All PA strains were cultured, identified, and analyzed using Microflex LT/SH (Bruker, Germany) and VITEK2 Compact (Mérieux, France). The susceptibility test results for 12 antibiotics [Ticarcillin/Clavulanic acid (TCC), imipenem (IPM), piperacillin (PIP), aztreonam (ATM), meropenem (MEM), ceftazidime (CAZ), cefoperazone/sulbactam (SCF), piperacillin/tazobactam (TZP), levofloxacin (LEV), ciprofloxacin (CIP), cefepime (FEP), and amikacin (AK)] referred to the M100 Performance Standards for Antimicrobial Susceptibility Testing (32nd edition) by the Clinical and Laboratory Standards Institute. The inclusion criteria for XDR-PA were that all 13 antibiotics were resistant except for colistin (COL). The study was approved by the Ethics Research Committee of Yunnan University Hospital (No. 2023009).

#### Multilocus sequence typing

We referred to the multilocus sequence typing (MLST) scheme for *P. aeruginosa* (PA) reported by Curran et al. (2004). First, seven genes of XDR-PA (acsA, aroE, guaA, mutL, nuoD, ppsA, and trpE) were amplified. The products were then sequenced and analyzed (Beijing Qingke Gene Technology Co., Ltd., China). The sequences were uploaded to a database (http://pubmlst.org/paeruginosa) for comparison, which allowed us to determine the type.

#### Arthrocolins isolation and characterization

Arthrocolins A, B, and C (Acs) were previously isolated from *Escherichia coli* using solvent extraction followed by chromatographic purification. Their chemical structures were determined based on nuclear magnetic resonance (NMR) spectroscopy and high-resolution mass spectrometry (HRMS), as reported in our earlier publication (Org. Lett. 2019, 21, 9745–9749). In this study, the purified compounds were directly used for antibacterial testing without further modification.

#### The drug susceptibility test

The drug susceptibility tests were conducted using the VITEK 2 COMPACT system (bioMérieux, France) following the guidelines of the Clinical and Laboratory Standards Institute (CLSI, M100, 32nd Edition, 2022). XDR-PA isolates were processed according to the manufacturer’s protocol, and minimum inhibitory concentrations (MICs) were automatically determined. Quality control was ensured using reference strains recommended by CLSI (e.g., *P. aeruginosa*ATCC 27853). The IC_50_ was determined according to the microbroth dilution method. XDR-PA was diluted to 1 × 10^5^ CFU/mL during the logarithmic phase of growth. A total of 100 μL of bacterial suspension and 1 μL of a specific concentration of Acs were added to each well of the microplate. The final concentrations of Acs were 0, 0.5 μM, 1.0 μM, 2.0 μM, 4.0 μM, and 8.0 μM, respectively, while the negative control received 1 μL of DMSO (Solarbio, China). The microplate was incubated at 30 °C in a 5% CO_2_ incubator for 24 h. The growth inhibition of XDR-PA by different concentrations of Acs was calculated by measuring the OD_600_ with a microplate reader (Thermo Fisher, United States).

Among these isolates, only one representative strain (ST3390) was used for IC_50_ determination, TEM observation, transcriptomic sequencing, and metabolomic profiling. The remaining strains were included only for epidemiological and antibiotic susceptibility analyses.

#### Transmission electron microscopy

All samples for transcriptomic, metabolomic, and TEM analyses were collected 20 h after Acs treatment to ensure comparability across datasets. XDR-PA was washed three times with sterile PBS solution, and the concentration was adjusted to 1 × 10^5^ CFU/mL. A total of 200 μL of fixing solution (2.5% glutaraldehyde) was added and incubated overnight at 4 °C. XDR-PA was washed three times with sterile PBS solution, then 100 μL of 1% osmium tetroxide solution was added and incubated at 4 °C for 2 h. After washing three times, graded concentrations of ethanol (5–100%) were used for dehydration, each for 15 min. Finally, the bacteria were treated with acetone solution for 5–10 min. The solution was drained, the bacteria were coated with Epon 812 resin, and ultra-thin slices with a thickness of 60 nm were cut using an EM UC7 microtome (Leica, Germany). The sections were observed with a JEM-1400 Transmission Electron Microscope (JEOL, Japan).

#### ATP level

Intracellular ATP levels were measured using the ATP content assay kit (Solarbio, China), following the manufacturer’s instructions. This kit utilizes a luciferase-based chemiluminescence assay to quantify ATP. The assay relies on the enzymatic reaction between ATP and luciferase, which produces light proportional to the ATP concentration. To measure ATP, bacterial cells were harvested and lysed to release intracellular ATP. The reaction mixture, which contains luciferase and other substrates, was then added to the sample. Luminescence was measured using a microplate reader (Model, Manufacturer), and ATP concentrations were determined by comparing the luminescence to a standard curve prepared with known ATP concentrations.

#### Intracellular lipid content detection

XDR-PA was washed three times with sterile PBS solution, and the concentration was adjusted to 1 × 10^5^ CFU/mL. One milliliter of the bacterial suspension was mixed with 500 μL of lipid fluorometric reagent (Abcam, UK) and incubated at 30 °C for 1 h. The strain was collected by centrifugation (5,000 rpm for 5 min) and washed three times with cold PBS solution. The fluorescence intensities were measured at excitation/emission wavelengths of 490/585 nm using flow cytometry (BD, United States), and the data were analyzed with FlowJo VX software.

#### Transcriptomic sequencing

All samples for transcriptomic, metabolomic, and TEM analyses were collected 20 h after Acs treatment to ensure comparability across datasets. Total RNA of XDR-PA was isolated using the TRIzol reagent. The sequencing library was sequenced on a NextSeq 500 platform (Illumina, United States) in Personal Biotechnology Co., Ltd. (Shanghai, China). RSEM software was used to quantify gene abundance. The differentially expressed genes were screened, and these differentially expressed genes were analyzed by GO and KEGG.

#### Metabolomics analysis

After treating XDR-PA under different conditions, the supernatant was collected. An untargeted metabolomics analysis of the supernatant samples was conducted at METABOLON’s Discovery HD4™ Metabolomics Platform [Calibra (DIAN) at Personal Biotechnology Co., Ltd. (Shanghai, China)]. Orthogonal Partial Least Squares Discriminant Analysis (OPLS-DA) was employed to determine discriminating metabolites using variable importance on projection (VIP). The *p*-value, VIP, and fold change were applied to identify contributing variables for classification. Finally, a *p*-value of 0.05 and VIP values greater than 1 were considered statistically significant for metabolites. Differential metabolites were subjected to pathway analysis using MetaboAnalyst, which combines results from powerful pathway enrichment analysis with pathway topology analysis. The identified metabolites in the metabolomics analysis were then mapped to the KEGG pathway for biological interpretation of higher-level systemic functions. The metabolites and corresponding pathways were visualized using the KEGG Mapper tool.

#### Statistical analysis

Data were processed using SPSS 21.0 and GraphPad Prism 8.0. Results are expressed as the mean ± standard deviation. The chi-square test was applied to analyze the clinical characteristics of the patients. A logistic regression model was used to analyze the correlation between hospitalization status and XDR-PA infection. A *t*-test was used for continuous variables. A *p*-value of < 0.05 was considered statistically significant, with * representing *p* < 0.05, ** representing *p* < 0.01, and *** representing *p* < 0.001.

## Results

Based on the methodologies described above, we next evaluated the antibacterial activity of arthrocolins (Acs) against XDR-*P. aeruginosa* and investigated their potential mechanisms of action.

### Clinical characteristics of patients with XDR-PA infection

Between December 2018 and June 2023, a total of 2,533 unique bacterial strains were isolated from The Affiliated Hospital of Yunnan University, excluding duplicate strains from the same patient. Among these, 250 strains were identified as XDR-PA. Clinical data analysis revealed that 228 cases were confirmed as XDR-PA infections, resulting in an XDR-PA detection rate of 9.0% (228/2,533). Of the XDR-PA-infected patients, 50.9% (116/228) had poor prognostic outcomes. To identify clinical risk factors for XDR-PA infection, we randomly included 228 patients with non-XDR-PA infections. The clinical characteristics of these patients are presented in Table 1. Notably, significantly fewer non-XDR-PA patients underwent antibiotic therapy, invasive procedures, clinotherapy, or required Intensive Care Unit (ICU) monitoring compared to XDR-PA patients (*p* < 0.001). Regression analysis identified the following as risk factors for XDR-PA infection: days of antimicrobial use (*p* = 0.019), length of stay (*p* < 0.001), clinotherapy > 1 month (*p* < 0.001), tracheal intubation (*p* = 0.004), and urinary catheterization (*p* < 0.001) (Table 2).

**Table 1:** Clinical characteristics of patients with non-XDR-PA or XDR-PA infection.

Characteristics	Non-XDR-PA (*n* = 228)	XDR-PA (*n* = 228)	*P*-value
Underlying disease			
Diabetes mellitus	39 (0.17)	44 (0.19)	0.544
Coronary heart disease	32 (0.14)	33 (0.14)	0.893
Antibiotic exposure			
Enzyme inhibitor	109 (0.47)	183 (0.80)	<0.001
Carbapenems	35 (0.15)	119 (0.52)	<0.001
Antifungal drugs	16 (0.07)	59 (0.26)	<0.001
Aminoglycosides	43 (0.19)	34 (0.15)	0.261
Quinolones	62 (0.27)	57 (0.25)	0.594
Invasive operation			
Tracheal intubation	66 (0.29)	197 (0.86)	<0.001
Intraurethral cannula	86 (0.38)	217 (0.95)	<0.001
Time			
Length of stay	15 (12, 19.5)	20.5 (15.5, 33.5)	<0.001
Days of antimicrobial treatment	11 (6.5, 16)	17 (12, 29)	<0.001
Patient status			
Clinotherapy > 1 month	78 (0.34)	198 (0.87)	<0.001
ICU	49 (0.21)	157 (0.69)	<0.001
Poor prognosis (death and discharge against medical advice)	35	116	<0.05

**Table 2:** Regression analysis of clinical characteristics with XDR-PA infection.

Factors	*P*-value	OR	95% CI
Days of antimicrobial use	0.019	0.936	(0.886, 0.989)
Length of stay	<0.001	1.094	(1.041, 1.150)
Clinotherapy > 1 month	<0.001	4.550	(2.405, 8.605)
Tracheal intubation	0.004	2.878	(1.395, 5.941)
Intraurethral cannula	<0.001	7.119	(3.138, 16.153)

OR, odds ratio; CI, confidence interval.

### Characteristics of PA and XDR-PA strains

To assess the drug susceptibility of *P. aeruginosa* (PA) strains, we analyzed their resistance to 12 antibiotics. The resistance rates of the 2,533 PA strains to these antibiotics were as follows: SCF (cefoperazone/sulbactam), 19.8%; PIP (piperacillin), 24.7%; TZP (piperacillin/tazobactam), 19.5%; CAZ (ceftazidime), 20.9%; FEP (cefepime), 11.1%; ATM (aztreonam), 24.1%; IPM (imipenem), 29.6%; MEM (meropenem), 21.4%; AK (amikacin), 7.1%; TCC (ticarcillin/clavulanic acid), 30.1%; CIP (ciprofloxacin), 13.3%; and LEV (levofloxacin), 15.2% (Table 3). These 228 clinical isolates were surveyed and characterized to determine epidemiological features and resistance patterns; however, they were not tested with arthrocolins (Acs). Instead, all subsequent IC_50_ determination and mechanistic studies were conducted using one representative strain, ST3390, which was selected for its clinical relevance and stable resistance phenotype.

**Table 3:** The antibiotics susceptibility test of PA and XDR-PA strains.

Antibiotics	PA (*n* = 2,533)		XDR-PA (*n* = 228)	
	R (%)	I (%)	R (%)	I (%)
SCF	19.8	9.2	97.2	2.8
PIP	24.7	14.1	100	0
TZP	19.5	15.2	96.4	3.6
CAZ	20.9	5.1	100	0
FEP	11.1	18.2	97.1	2.9
ATM	24.1	22.2	98.3	1.7
IPM	29.6	0	98.9	1.1
MEM	21.4	5.1	95.4	4.6
AK	7.1	0	96.5	3.5
TCC	30.1	13.4	100	0
CIP	13.3	2	100	0
LEV	15.2	8.1	100	0

R, resistance rate; I, intermediate rate.

### Antibacterial effect of Acs on XDR-PA

**Figure 1:**
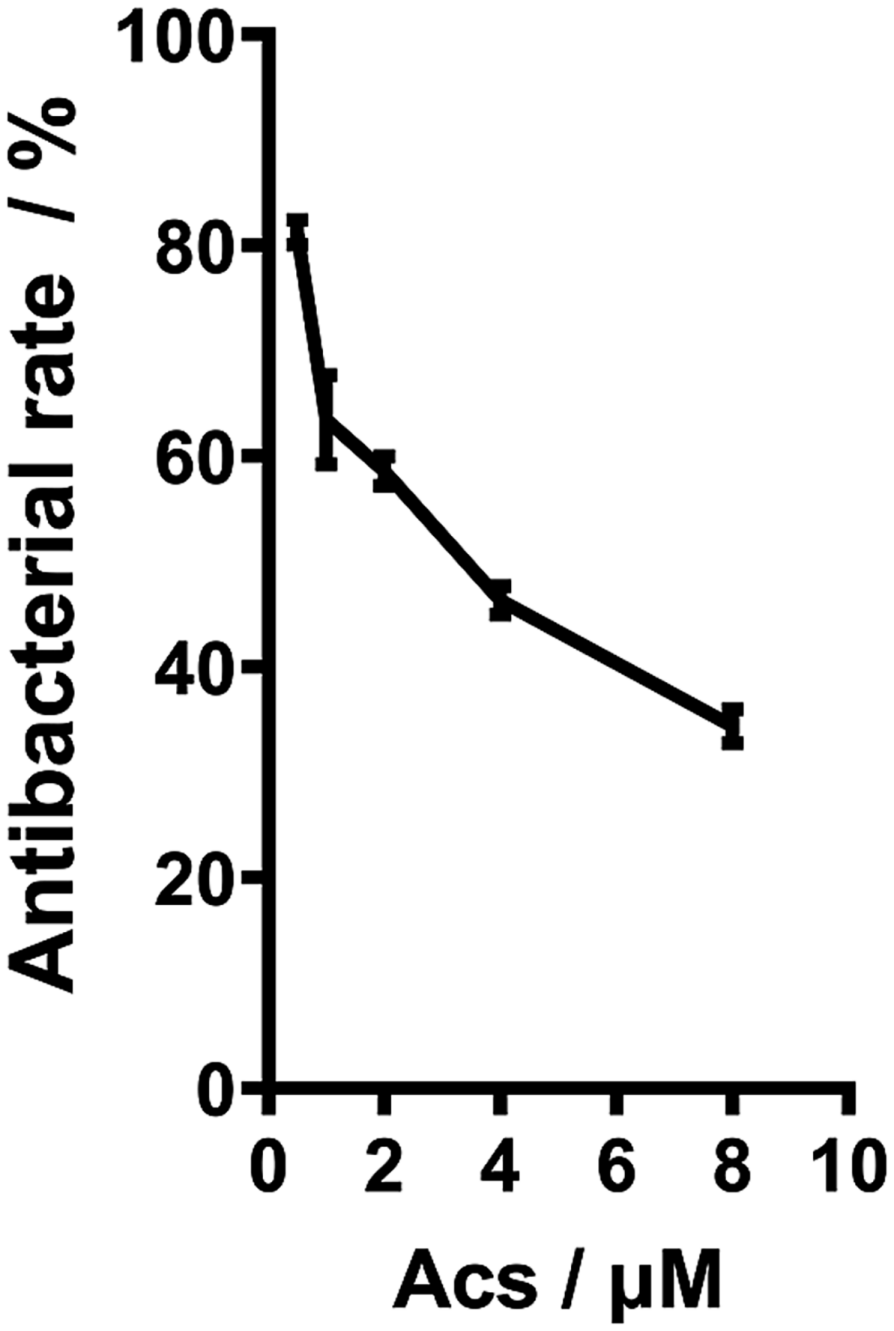
The antibacterial effect of Acs on XDR-PA (*n* = 3). XDR-PA cells were treated with varying concentrations of Acs (0, 5, 10, and 20 μM) for 24 h. The antibacterial effect was assessed by measuring bacterial growth inhibition using a microplate reader at 600 nm. Data represent the mean ± standard deviation (SD) of three independent experiments (*n* = 3). Statistical significance was analyzed using a one-way ANOVA (*p < 0.05).

The chemical structures of arthrocolins A–C have been fully characterized in our previous work (Org. Lett. 2019, 21, 9745–9749) based on NMR and HRMS analyses. As these structures have been repeatedly reported, they are not duplicated here, and readers are referred to our earlier publication for detailed structural representations. The ST3390 subtype is the predominant carbapenem-resistant PA strain isolated from local hospitals. In this study, we selected an XDR-PA strain with the MLST subtype ST3390 as a representative to investigate the antibacterial effect of Acs. This XDR-PA strain was isolated from the urine of a hospitalized patient. Drug susceptibility testing revealed that Acs was tested at concentrations ranging from 0 to 10 μM (Figure 1), within which the effective inhibitory concentrations of 0.5–8 μM were used for IC_50_ determination, yielding a value of 3.094 μM (Figure 1). It should be noted that all IC_50_ measurements and subsequent mechanistic studies described in this work were conducted using this representative ST3390 strain, whereas the other 228 clinical isolates were only included for epidemiological and antibiotic susceptibility characterization. Additionally, the antibacterial activity of Acs was compared to that of commonly used antibiotics, including meropenem and ceftazidime, which are standard treatments for carbapenem-resistant *P. aeruginosa*. Acs demonstrated comparable or superior activity at lower concentrations (1–8 μM) compared to these antibiotics, which required higher concentrations to achieve similar effects. These results highlight the potential of Acs as an effective alternative for treating XDR-PA infections, particularly in cases where standard treatments fail.

### Acs caused metabolic disorders in XDR-PA

**Figure 2:**
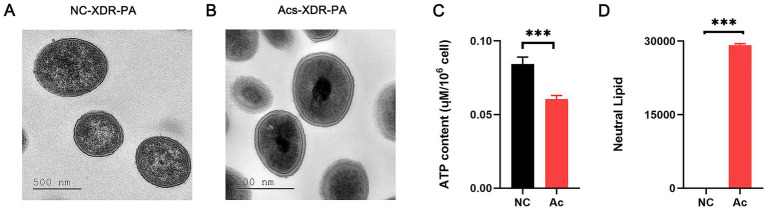
**(A,B)** TEM imaging of the changes in the basal structure of XDR-PA induced by NC and Acs treatment. Scale bars: 500 nm. **(C)** The level of ATP produced in XDR-PA. **(D)** The neutral lipid level in XDR-PA. NC group: XDR-PA untreated with Acs (NC group as the negative control). Acs group: XDR-PA treated with 3.094 μM Acs (*n* = 3). *** representing *p* < 0.001.

We observed the changes in the basal structure of XDR-PA using TEM. The imaging showed that XDR-PA without Acs treatment had an intact cell wall and abundant cytoplasm (Figure 2A). However, after treatment with 3.094 μM Acs, the cell wall appeared blurred, and the cytoplasmic material was visibly aggregated into clusters, with no clear organelle distribution (Figure 2B). Additionally, we observed a significant reduction in ATP levels (*p* < 0.001) and excessive lipid accumulation (*p* < 0.001) in XDR-PA strains treated with 3.094 μM Acs, as shown in Figures 2C,D.

### Transcriptomic sequencing and analysis

**Figure 3:**
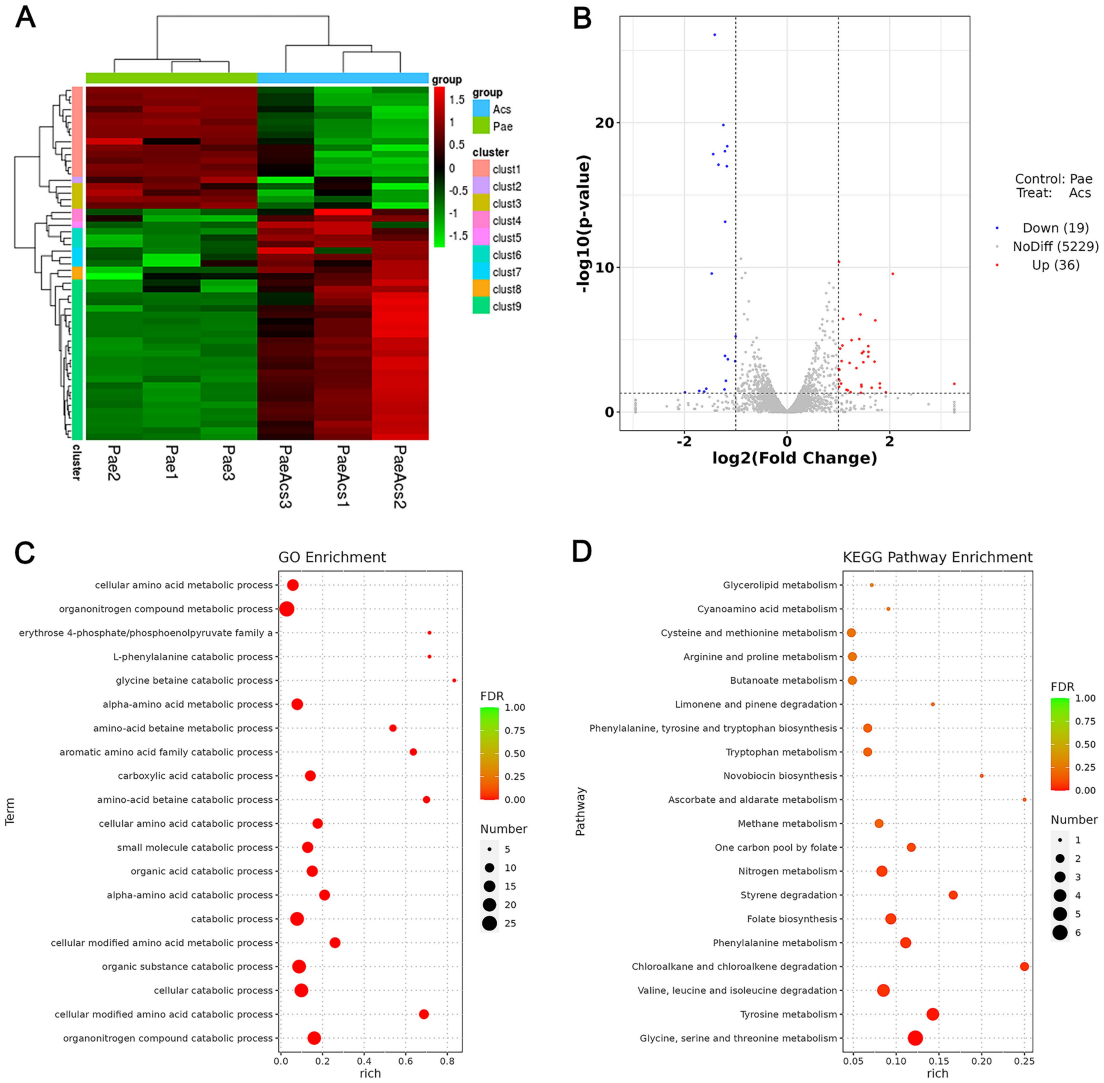
Transcriptomic sequencing and differential expression analysis (Pae group as the baseline control). **(A)** Cluster analysis: The heatmap shows the statistical trend of differentially expressed genes in the Pae and Acs groups. Each column represents one sample, and each row represents one gene. Red indicates up-regulated genes, green indicates down-regulated genes, and black indicates non-differentially expressed genes. **(B)** Volcano plot: The abscissa is log_2_ (Fold Change), and the ordinate is -log_10_ (*p*-value). The two vertical dotted lines represent the 2-fold expression difference threshold, and the horizontal dotted line represents the *p*-value = 0.05 threshold. Red dots indicate up-regulated genes, blue dots indicate down-regulated genes, and gray dots indicate non-significantly differentially expressed genes. **(C)** GO enrichment analysis bubble plot. **(D)** KEGG enrichment analysis bubble plot: Pae group vs. Acs group (*n* = 3).

To explore the potential antibacterial mechanism of Acs in inhibiting XDR-PA, we analyzed the transcriptomes of bacterial strains under two conditions: the Pae group (XDR-PA untreated with Acs) and the Acs group (XDR-PA treated with 3.094 μM Acs). Clustering analysis revealed differential gene expression between the Pae and Acs groups, with some genes showing opposite trends (Figure 3A). The volcano plot indicated 36 up-regulated genes and 19 down-regulated genes in the Acs group (Figure 3B). Gene Ontology (GO) enrichment analysis of these differentially expressed genes identified the top five significant pathways in the Acs group: organonitrogen compound catabolic process, cellular modified amino acid catabolic process, cellular catabolic process, organic substance catabolic process, and cellular modified amino acid metabolic process (Figure 3C). Kyoto Encyclopedia of Genes and Genomes (KEGG) enrichment analysis highlighted the most enriched pathways in the Acs group, including glycine, serine and threonine metabolism; tyrosine metabolism; valine, leucine and isoleucine degradation; chloroalkane and chloroalkene degradation; and phenylalanine metabolism (Figure 3D). These findings suggest that Acs primarily affects the amino acid metabolic pathway in XDR-PA. Amino acid metabolism plays a critical role in bacterial energy production, cellular component synthesis, and the maintenance of internal environmental stability.

### Characteristics of metabolomics

**Figure 4:**
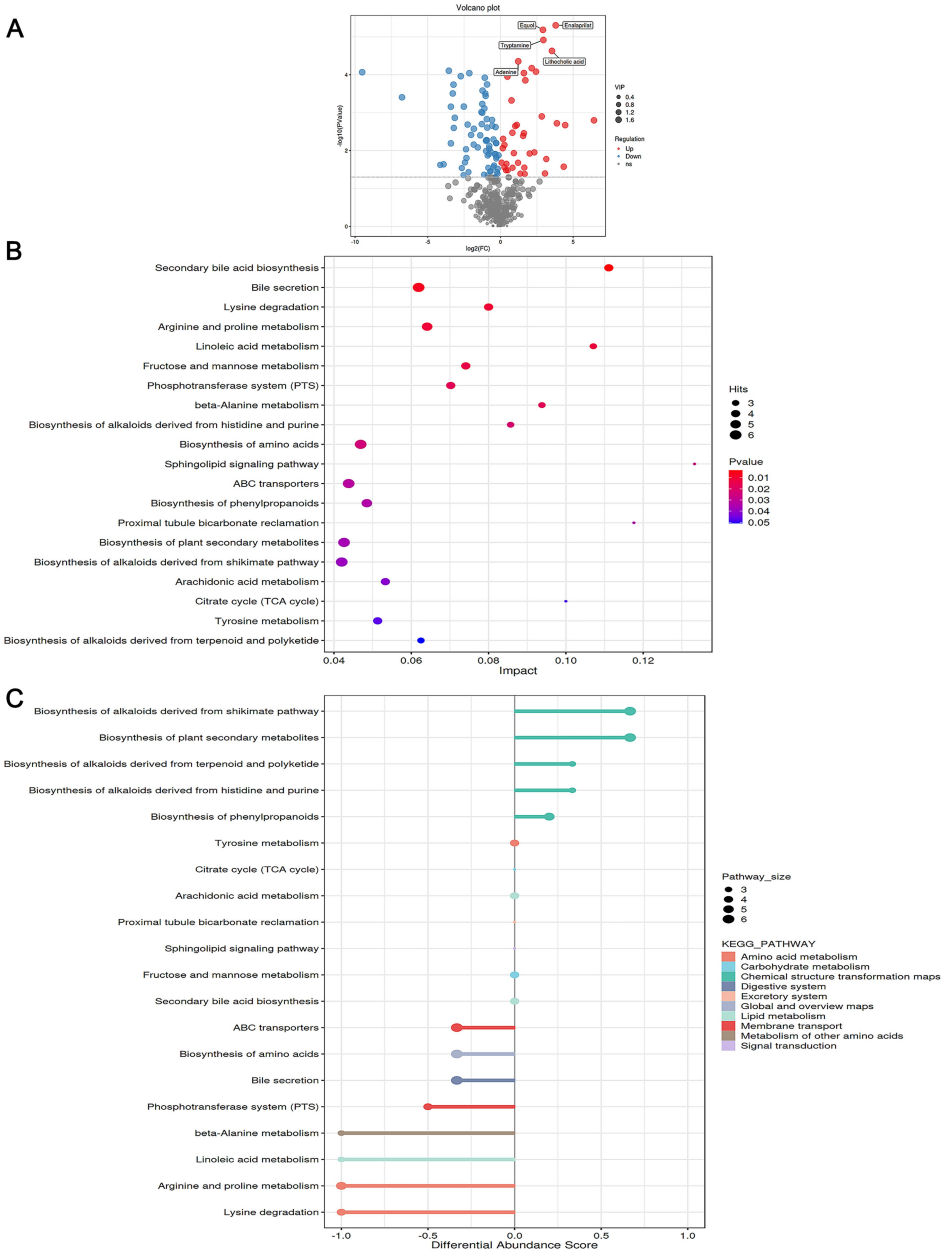
Metabolomics analysis (Pae group as the baseline control). **(A)** Volcano plot: The abscissa is log_2_ (Fold Change), and the ordinate is −log_10_ (*p*-value). Red dots represent up-regulated metabolites, blue dots represent down-regulated metabolites, and gray dots represent non-significantly differentially expressed metabolites. **(B)** KEGG enrichment analysis bubble plot. **(C)** Correlation analysis among the metabolic pathways based on KEGG enrichment analysis: *Pae* group vs. Acs group (*n* = 3).

Using non-targeted metabolomics, we further assessed the impact of Acs treatment on XDR-PA metabolism. A total of 460 metabolites were identified, with 108 metabolites showing differential expression after computational screening (*p* < 0.05). Among these, 39 metabolites were up-regulated, while 69 were down-regulated in the Acs group compared to the Pae group (Table 4; Figure 4A). Pathway analysis of these differential metabolites identified the top five KEGG-enriched pathways with significant changes: sphingolipid signaling pathway, proximal tubule bicarbonate reclamation, secondary bile acid biosynthesis, linoleic acid metabolism, citrate cycle, and beta-alanine metabolism (Figure 4B). Additionally, correlation analysis of the metabolic pathways revealed that chemical structure transformation maps, amino acid metabolism, and membrane transport exhibited high differential abundance scores and a large number of associated metabolites (Figure 4C).

**Table 4:** The top 5 metabolites were up-regulated and down-regulated expressed in the Acs group.

Metabolites	Foldchange	*P*-value	VIP	Trend
Triethylamine	86.44	0.001595419	1.591181005	Up
UMP	21.89	0.002136002	1.611024262	Up
Genistein	20.41	0.026630418	1.562168878	Up
12-KETE	14.69	0.001908606	1.63916617	Up
Enalaprilat	13.96	4.99393E-06	1.671309879	Up
Maleic acid	0	8.59168E-05	1.66293799	Down
3-Epiecdysone	0.01	0.000393757	1.619219312	Down
Suberic acid	0.06	0.023850631	1.449409402	Down
2,3-Dinor-8-iso prostaglandin F2alpha	0.07	0.023036473	1.565308788	Down
L-Gulose	0.09	0.000697743	1.640034112	Down

## Discussion

In this study, fluorescein-like arthrocolins (Acs) displayed concentration-dependent antibacterial activity against an XDR *Pseudomonas aeruginosa* clinical isolate and induced marked metabolic and structural perturbations. Integrating phenotypic readouts with our omics results supports a model in which Acs primarily disrupt amino acid metabolism, with downstream consequences for energy production and cell integrity, thereby compromising bacterial viability. Clinically, such a mechanism could be relevant to high-risk populations in whom resistant *P. aeruginosa* infections are associated with adverse outcomes—e.g., patients with hematologic malignancies and other immunocompromised cohorts, as well as pediatric populations (Ruhnke et al., 2014; Trecarichi et al., 2014; Yang et al., 2011).

While detailed structural exposition is provided elsewhere in the manuscript, several features of Acs are mechanistically consistent with the present findings. Their fluorescein-like scaffold and functional groups may enable redox interactions and engagement with metabolic enzymes, offering a plausible structural basis for the observed targeting of amino-acid–linked pathways. These considerations align with previously reported pleiotropic bioactivities of Acs in non-bacterial systems and rationalize their capacity to reprogram metabolism at bactericidal concentrations.

Multiple lines of evidence converge on amino-acid–centered metabolic vulnerability. First, branched-chain amino acids (BCAAs) are tightly coupled to ATP generation via glucose transporter translocation, so perturbing BCAA availability or flux can reduce cellular energy charge (Iwai et al., 2022). Second, amino acid metabolism is fundamental to organelle and cellular homeostasis; its disruption propagates stress responses and bioenergetic deficits (Li and Hoppe, 2023; Wu, 2009). Third, interference with the tricarboxylic acid (TCA) cycle integrates redox and amino acid signaling through conserved stress axes (e.g., ATF4-dependent programs), conceptually explaining how metabolic imbalance scales into global dysfunction (Ryan et al., 2021). Beyond L-amino acids, D-amino acids also serve structural and regulatory roles in microbial communities, and their pools are sensitive to metabolic rewiring (Aliashkevich et al., 2018). Finally, carbon source selection can tune antibiotic susceptibility via TCA control, indicating that central metabolism is a master regulator of drug response in *P. aeruginosa* (Meylan et al., 2017). Together, these data provide a coherent rationale for why Acs-driven interference with amino acid metabolism would lower energy availability, impair biosynthesis, and culminate in loss of viability.

In addition to amino acids, our datasets indicated alterations in sphingolipid-related intermediates. Although the bacterial sphingolipid repertoire is limited compared with eukaryotes, shifts in lipid intermediates can influence membrane organization, redox balance, and signaling. Such changes plausibly “talk” to amino acid pathways by (i) altering the cellular redox state that governs amino acid catabolism and anaplerosis and (ii) modulating membrane-embedded transport/enzymatic steps that set amino acid influx/efflux. This crosstalk offers a parsimonious explanation for the coordinated metabolic and structural phenotypes we observed and merits targeted follow-up experiments.

Current therapies for XDR *P. aeruginosa* remain imperfect in populations with high morbidity and mortality risk (Ruhnke et al., 2014; Trecarichi et al., 2014; Yang et al., 2011). Although newer β-lactam/β-lactamase inhibitor combinations such as ceftolozane–tazobactam have expanded options, activity is heterogeneous across resistance backgrounds (Shortridge et al., 2018). By contrast, Acs appear to act primarily via metabolic derailment rather than classical targets (cell wall, ribosome, or DNA), suggesting a complementary mechanism that could (i) bypass established resistance determinants and (ii) potentiate combination strategies with existing agents.

Proteome-level remodeling is a hallmark of stress and drug exposure in *P. aeruginosa*. Antimicrobial peptides such as Actifensin and Defensin-d2 induce broad proteomic shifts in MDR isolates, underscoring the therapeutic potential of agents that rewire bacterial physiology (Gbala et al., 2022). Likewise, under iron starvation, the PrrF sRNA network controls proteins involved in twitching motility, amino acid metabolism, and metal homeostasis, illustrating how regulatory RNAs coordinate metabolic and virulence programs (Nelson et al., 2019). Recent reviews synthesize these themes, outlining novel therapeutic strategies against *P. aeruginosa* (Qin et al., 2022) and explored the convergent within-host adaptation of Pseudomonas aeruginosa through its transcriptional regulatory network (Gatt et al., 2023; Zhao et al., 2024). Placing our findings within this framework supports the concept that targeting metabolic vulnerabilities—particularly amino acid–linked nodes—may be an effective route to overcome multidrug resistance.

Considering the clinical stakes, emerging modalities—including phage therapy, antimicrobial peptides, and anti-virulence agents—are being actively developed as complements to antibiotics (Dehbanipour and Ghalavand, 2022). Given their distinct, metabolism-centric action, Acs warrant evaluation in rational combinations (e.g., with agents whose uptake or activity is enhanced by increased metabolic demand) and across diverse, high-risk clones to assess spectrum, resistance suppression, and translational potential.

### Limitations of the study

This work has limitations. First, the precise molecular targets of Acs remain unidentified; biochemical target-engagement assays and structural studies are needed to define direct binding partners. Second, our evaluation focused on a single XDR isolate; validation across additional high-risk clones (e.g., ST235) is required to assess generalizability. Third, the absence of *in vivo* data limits translational inference; animal infection models will be essential to establish efficacy and safety. Fourth, potential cytotoxicity of Acs toward mammalian cells was not addressed here and should be systematically profiled. These limitations delineate clear next steps toward mechanism elucidation and preclinical development.

In conclusion, our findings establish Acs as promising metabolic disruptors with potent antibacterial activity against XDR *P. aeruginosa*. By linking chemical structure to metabolic vulnerability, we provide mechanistic evidence that interference with amino acid–centered pathways can undermine bacterial viability. Placing these results in the broader context of resistance regulation and emerging therapeutic strategies highlights the translational potential of Acs. Future work addressing the limitations noted above will be essential to advance Acs toward preclinical development and integration into the expanding arsenal against multidrug-resistant pathogens.

## Data Availability

The raw data from the transcriptomic experiment have been deposited in the SRA database of NCBI under accession number PRJNA1127659446 (https://www.ncbi.nlm.nih.gov/guide/). The raw data from the metabolomic experiment can be found in Metabolights under accession number MTBLS10539448 (https://www.ebi.ac.uk/metabolights/editor/login).
